# Isoforms of endothelin-converting enzyme-1 (ECE-1) have opposing effects on prostate cancer cell invasion

**DOI:** 10.1038/sj.bjc.6604631

**Published:** 2008-09-09

**Authors:** L A Lambert, A R Whyteside, A J Turner, B A Usmani

**Affiliations:** 1 Proteolysis Research Group, Institute of Molecular and Cellular Biology, Faculty of Biological Sciences, University of Leeds, Leeds LS2 9JT, UK

**Keywords:** Endothelin-converting enzyme-1 (ECE-1), cell invasion, neprilysin, suppressor, prostate cancer

## Abstract

Cross-talk between tumour and stromal cells can profoundly influence cancer cell invasion by increasing the availability of mitogenic peptides such as endothelin-1 (ET-1). Endothelin-1 is elevated in men with metastatic prostate cancer (PC), and can exert both an autocrine (epithelial) and a paracrine (stromal) influence on growth. Endothelin-1 is generated from its inactive precursor big-ET-1 by endothelin-converting enzyme 1 (ECE-1). We and others have demonstrated that ECE-1 expression is significantly elevated in tumours and surrounding stromal tissue. Our current data show siRNA-mediated knockdown of stromal ECE-1 reduces epithelial (PC-3) cell invasion in coculture. Interestingly, readdition of ET-1 only partially recovers this effect suggesting a novel role for ECE-1 independent of ET-1 activation. Parallel knockdown of ECE-1 in both stromal and epithelial compartments results in an additive decrease in cell invasion. We extrapolated this observation to the four recognised isoforms ECE-1a, ECE-1b, ECE-1c and ECE-1d. Only ECE-1a and ECE-1c were significant but with reciprocal effects on cell invasion. Transient ECE-1c overexpression increased PC-3 invasiveness through matrigel, whereas transient ECE-1a expression suppressed invasion. Furthermore, transient ECE-1a expression in stromal cells strongly counteracts the effect of transient ECE-1c expression in PC-3 cells. The ECE-1 isoforms may, therefore, be relevant targets for antiinvasive therapy in prostate and other cancers.

Alteration of interactions between the epithelial and stromal compartments can result in a loss of tissue homeostasis and the induction of malignant epithelia. This, in turn, influences adjacent stroma to generate a dynamically altered environment providing factors for growth (Yang *et al*, 2005; Albini and Sporn, 2007). One such factor, endothelin-1 (ET-1), is evident in prostatic tissue *ex vivo* (Godara *et al*, 2007). Endothelin-1 is abnormally elevated in men with advanced metastatic prostate cancer (PC) and contributes to the transition to androgen independence (Nelson *et al*, 1995; Papandreou *et al*, 1998). Endothelin-1 is generated from its inactive precursor big ET-1 by endothelin-converting enzyme 1 (ECE-1). We and others have shown that ECE-1 expression is significantly elevated in tumours (Ahmed *et al*, 2000; Eberl *et al*, 2000; Egidy *et al*, 2000b; Arun *et al*, 2001; Usmani *et al*, 2002; Dawson *et al*, 2004, 2006; Awano *et al*, 2005; Smollich *et al*, 2007). We have previously demonstrated that ECE-1 is present in PC cell lines and primary tissues, and is expressed at the cell surface and intracellularly (Dawson *et al*, 2004, 2006), with levels of ECE-1 elevated in primary malignant stromal cells as compared with benign (Dawson *et al*, 2004). The specific inhibition of endogenous ECE-1 activity in these stromal cells significantly reduced epithelial cell invasion (Dawson *et al*, 2004).

Endothelin-converting enzyme 1 is a membrane-bound zinc metalloprotease composed of a large extracellular or luminal C-terminal catalytic domain, a transmembrane region and a small N-terminal cytoplasmic domain. There are four distinct ECE-1 isoforms; ECE-1a (758 residues), ECE-1b (770) (Shimada *et al*, 1995); ECE-1c (754) (Schweizer *et al*, 1997) and ECE-1d (767) (Valdenaire *et al*, 1999). These isoforms differ only in part of their N-terminal cytoplasmic regions and are derived from a single gene through the use of alternative promoters (Orzechowski *et al*, 1997). They have similar catalytic properties but distinct subcellular localisation and tissue distribution (Schweizer *et al*, 1997). Endothelin-converting enzyme 1a and ECE-1c are localised at the cell surface, whereas ECE-1b and ECE-1d are intracellular with ECE-1b present in late endosomes or multivesicular bodies and ECE-1d concentrated in recycling endosomes (Muller *et al*, 2003). More recently, ECE-1 has been reported to regulate peptide receptor recycling in endosomes through metabolism of the peptide ligand (Roosterman *et al*, 2007). Endothelin-converting enzyme 1c is the major isoform in terms of expression level and tissue distribution (Schweizer *et al*, 1997). We have shown that ECE-1c is the main isoform expressed in prostate epithelial cells, with increased expression in metastatic, androgen-independent cell lines (Dawson *et al*, 2006). ECE-1c is also the most abundant isoform in lung cancer-derived cell lines (Ahmed *et al*, 2000).

Endothelin-converting enzyme 1 shares 40% homology with a fellow member of the M13 family of endopeptidases, neprilysin (NEP). Neprilysin is downregulated in metastatic human PC and contributes to the transition to the androgen-independent disease (Papandreou *et al*, 1998) both through catalytic and non-catalytic signalling interactions. Neprilysin can affect signal transduction pathways, which regulate cell migration (Sumitomo *et al*, 2000) apoptosis (Sumitomo *et al*, 2004) and Akt-mediated survival (Sumitomo *et al*, 2001), and has also been reported to be a tumour suppressor (Dai *et al*, 2001) directly associating with PTEN (Sumitomo *et al*, 2004). Signalling capability, therefore, is already recognised among the M13 family.

In this study, we have investigated the invasion of metastatic epithelial cells with regard to ECE-1 isoform expression, and herein provide the first evidence for distinct non-catalytic roles for two of the ECE-1 isoforms, ECE-1a and ECE-1c, in promoting and suppressing PC cell invasion, mediated through their unique N-terminal regions.

## Materials and methods

### Materials

The ECE-1 monoclonal antibody AEC 32-236, as described by (Shimada *et al*, 1994) was generously donated by Dr K Tanzawa (Sankyo Research Laboratories, Tokyo, Japan). The polyclonal antibody against the ECE-1c isoform is produced in-house as described and characterised previously (Brown *et al*, 1998). Primary prostate cells and the PNT1-a cell line were kindly provided by Prof Norman Maitland, York Cancer Research Unit, UK. Matrigel, cell culture inserts and companion plates were obtained from BD Biosciences (Bedford, UK). The siGLO™ RISC-Free siRNA (non-targeting siRNA with a fluorescent label) was purchased from Dharmacon via Perbio, Cramlington, UK. The individual siRNA duplex targeting the ECE-1 sequence (CTTCCACAGCCCCCGGAGT), common to all ECE-1 isoforms, and the scramble (nonsense) control was custom synthesised by Dharmacon. The pool siRNA containing four different oligonucleotides targeted to the ECE-1 sequence was also synthesised by Dharmacon. Endothelin-1 was purchased from Calbiochem (Nottingham, UK).

### Methods

*Cell culture* The PC-3 cells were routinely cultured in Ham's F12 nutrient mix (BioWhittaker, Wokingham, UK) supplemented with 2 mM L-glutamine and 7% (v/v) FBS. The PNT1-a cells and the primary prostate stromal cells were maintained in RPMI-1640 (BioWhittaker, Wokingham, UK) containing 2 mM L-glutamine and 10% (v/v) FBS. Penicillin at 1% (v/v) and streptomycin (50 U ml^−1^) were added to the primary stromal cell media. STO (mouse embryonic fibroblast) cells were routinely cultured in Dulbecco's modified Eagle's medium (DMEM; BioWhittaker, Wokingham, UK) containing 2 mM L-glutamine and 10% (v/v) FBS. All cells were routinely grown in antibiotic-free media at 37°C and 5% CO_2_.

*Construction of ECE-1 isoform expression plasmids* Endothelin-converting enzyme 1 isoforms were amplified from EA.hy926 (epithelial/endothelial hybrid cell line) RNA. Each isoform was amplified by one-step RT-PCR using an isoform-specific forward primer incorporating a unique *Not* 1 restriction site and a common reverse primer incorporating a unique *Xba* 1 restriction site. The amplified products were digested and ligated into the pcDNA3 mammalian expression vector (Invitrogen, Paisley, UK). Each construct was fully sequenced for verification.

*Lipid-mediated oligonucleotide transfection into adherent mammalian cells* Cells were seeded at 50% confluency and transfected with siRNA duplexes (Dharmacon) using Oligofectamine™ (Invitrogen, Paisley, UK) according to the manufacturer's guidelines. Cells were transfected with 100 nM duplexes targeted to ECE-1 or the control duplex siGLO™.

*Transient transfection of ECE-1 isoforms* Cells at 60% confluency were transfected with ECE-1a, ECE-1b, ECE-1c or ECE-1d expression plasmids using FuGENE-6 (3 : 2 ratio of FuGENE-6 to DNA; Roche, UK) according to the manufacturer's instructions.

*Western blot analysis* Protein was isolated from whole cell lysates and resolved by SDS–PAGE, transferred to a nitrocellulose membrane and blocked in 0.1% Tween-20 in 10 mM Tris-HCl, pH 7.4 (TBST) with 5 % (w/v) milk powder and 2% (w/v) BSA. Membranes were incubated with anti-ECE-1 monoclonal (1 : 500) or anti-ECE-1c polyclonal (1 : 500). Anti-*β*-actin (Sigma, Pool, UK; 1 : 10 000) was used as a loading control. Immunoreactive bands were visualised using enhanced chemiluminescence (ECL).

*Immunofluorescence* Cells were grown to 60% confluency on sterile coverslips, washed twice with PBS and fixed and permeabilised for 10 min in methanol/acetone (1 : 1 ratio) at room temperature. Non-specific binding sites were blocked for 30 min in blocking buffer (TBS, 1% (v/v) normal goat serum and 0.2% (w/v) gelatin). Primary antibodies were used at the following concentrations: ECE-1 monoclonal (1 : 50) and ECE-1c (1 : 100). For negative controls, the primary antibody was replaced with preimmune serum or IgG subclass antibody (Sigma, Poole, UK). Cells were incubated for 30 min at room temperature with FITC-conjugated anti-mouse IgG (1 : 1000) or FITC-conjugated anti-rabbit IgG (1 : 1000, Jackson ImmunoResearch Laboratory via Stratech Scientific, Newmarket, UK) and counterstained using 4′,6-Diamidino-2-phenylindole (DAPI; Sigma, Poole, UK). Cells were examined using an Olympus IX70 inverted wide-field fluorescence microscope. Images were captured using Delta Vision from Applied Precision.

*Invasion assay* The invasion assay was performed essentially as described by Dawson *et al*., (2004). Briefly, invasion chambers were prepared using matrigel (250 *μ*g ml^−1^), which was added (200 *μ*l) to cell culture inserts (8 *μ*m pore) and incubated overnight at 37°C. In parallel, STO cells or primary prostate stromal cells were seeded into 24-well companion plates and incubated overnight at 37°C. On the following day, matrigel-coated inserts were placed in the wells of the companion plates and PC-3 cells (2 × 10^5^) in DMEM 0.1% (w/v) BSA were added to the insert. When ET-1 supplements were required, they were added to both STO/stromal cell media (companion plate) and epithelial cell media (insert). The invasion assay was incubated overnight at 37°C. Next day, the inserts were removed from the wells, washed in PBS, fixed in 100% methanol for 10 min at room temperature and stained with 0.1% (w/v) crystal violet (Sigma, Poole, UK). Cells that had invaded to the underside of the inserts were counted by light microscopy. Four fields of view from each insert were counted. The data distribution was shown to be normal using the SPSS NPAR test, and therefore two-tailed student T-tests were used to ascertain statistical significance with a threshold of *P*<0.05.

## Results

### Endothelin-converting enzyme 1 knockdown in either stroma or epithelia can reduce cell invasion

**Figure 1:**
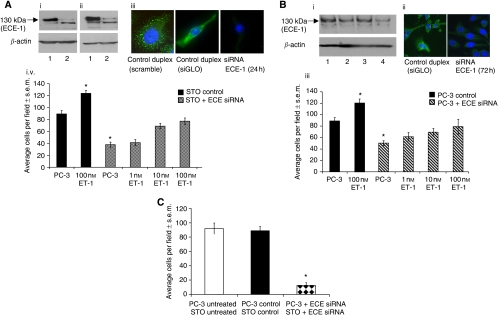
(**A**) Influence of ECE-1 siRNA expressed in stromal cells. STO cells were transfected with 100 nM ECE-1 siRNA oligonucleotides targeted to ECE-1 (either custom-made duplexes or a pool containing four different target sequences to eliminate the possibility of off-target effects) or oligonucleotides with a non-coding sequence (siGLO or scrambled sequence) using oligofectamine and incubated for 24 h. Samples were analysed using (i) western blotting: lane 1, siGLO-treated STO cells; lane 2, custom-made siRNA duplex to ECE-1; (ii) lane 1, siGLO-treated STO cells; lane 2, siRNA ECE-1 pool or (iii) immunofluorescence analysis. The polyclonal antibody to ECE-1 was used for the western blot analysis (1 : 500) and immunofluorescence (1 : 100). Anti-*β*-actin (1 : 10,000) was used as a loading control for western blotting. (iv) Invasion of PC-3 cells was measured in the presence of the ECE-1 siRNA-treated STO cells using a matrigel invasion assay. Endothelin-converting enzyme 1 siRNA STO cells were harvested 24 h post-transfection and added to the lower well of the invasion chamber. Endothelin-1- at 1–100 nM was added to both the STO (lower well) and the PC-3 (upper well), and the assay was incubated for 24 h. Each bar represents the mean value of eight fields counted; ^*^P<0.001. (**B**) Influence of ECE-1 siRNA expressed in epithelial cells. The PC-3 cells were transfected with 100 nM ECE-1 siRNA using oligofectamine and incubated 24–72 h. siGLO siRNA was used as a negative control. Samples were analysed using (i) western blotting: lane 1, siGLO-treated STO cells; lanes 2–4, siRNA ECE-1 24–72 h or (ii) immunofluorescence analysis. The monoclonal antibody to ECE-1 was used for western blot analysis (1 : 200) and immunofluorescence (1 : 50). Anti-*β*-actin (1 : 10 000) was used as a loading control for western blotting. (iii) Invasion of ECE-1 siRNA PC-3 cells was measured in the presence of the STO cells using a matrigel invasion assay. Endothelin--1 at 1–100 nM was added to both the STO (lower well) and the PC-3 (upper well), and the assay was incubated for 24 h. Each bar represents the mean value of eight fields counted; ^*^P<0.001. (**C**) Influence of ECE-1 siRNA expressed in both stromal and epithelial cells. Both the PC-3 cells and the STO cells were treated with siRNA ECE-1 and added to a matrigel invasion assay. Each bar represents the mean value of eight fields counted; ^*^P<0.001.

To determine the effect of stromal ECE-1 depletion on invasion, STO cells were transfected with either 100 nM siRNA duplex oligonucleotides targeted to ECE-1 (either custom-made duplexes or a pool containing four different target sequences to eliminate the possibility of off-target effects) or oligonucleotides with a non-coding sequence (siGLO or scrambled sequence). The protein expression of ECE-1 was measured at 24 h by western blotting and immunofluorescent analyses. Endothelin-converting enzyme 1 levels decreased 24 h post-transfection (Figure 1A (i), (ii) and (iii)). In siGLO or scramble transfected cells, no change was observed. The influence of ECE-1 siRNA-treated STO cells, in coculture, on PC-3 invasion through matrigel was subsequently analysed (Figure 1A (iv)). STO cells harbouring siGLO had no significant effect on PC-3 cell invasion. STO cells harbouring ECE-1 siRNA caused a decrease to approximately 40% of control levels in PC-3 cell invasion. Supplementation with ET-1 (1–100 nM) recovered cell invasion maximally to approximately 85% of control levels (Figure 1A (iv)). In PC-3 cells, ECE-1 protein expression was analysed by western blotting and immunofluorescence following transfection with ECE-1 siRNA over a period of 72 h. Endothelin-converting enzyme 1 expression visibly decreased 72 h post-transfection (Figure 1B (i) and (ii)). The influence of ECE-1 depletion in the epithelial compartment (PC-3), in coculture with untreated STO cells, was subsequently measured (Figure 1B (iii)). The invasion of ECE-1-depleted PC-3 cells decreased by approximately 50%, when compared with control PC-3 cells (transfected with siGLO). Supplementation with 1–100 nM ET-1 recovered invasion maximally to approximately 85% of control levels. To examine the effect of ECE-1 depletion in both epithelial and stromal compartments, the invasion of ECE-1-depleted PC-3 cells was measured in the presence of ECE-1-depleted STO. Invasion decreased to less than 20% of control levels (Figure 1C).

### Endothelin-converting enzyme 1a and ECE-1c isoforms suppress and promote invasion, respectively

**Figure 2:**
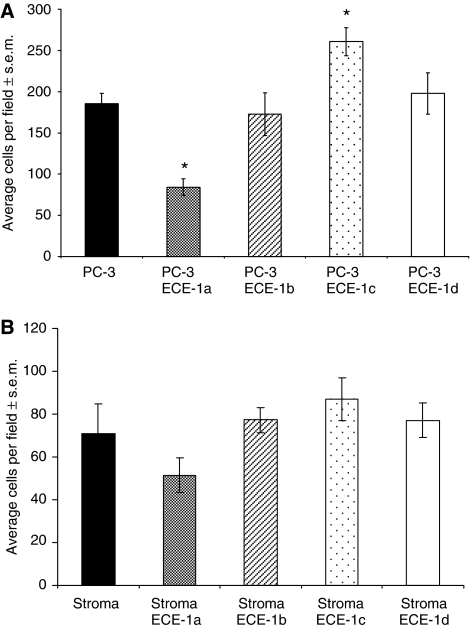
The effect of ECE-1 isoform expression in either the epithelial or stromal compartment on invasion (**A**) PC-3 cells were transiently transfected with ECE-1a, ECE-1b, ECE-1c and ECE-1d using FuGENE-6 transfection reagent. Following a 48-h incubation, the cells were harvested and used in a matrigel invasion assay in the presence of stromal cells. Each bar represents the mean value of eight fields counted; ^*^P<0.001. (**B**) Stromal cells were transfected with ECE-1a ECE-1b, ECE-1c and ECE-1d using FuGENE-6 and incubated for 48 h. Invasion of PC-3 cells was measured in the presence of the ECE-1-transfected stromal cells using a matrigel invasion assay. Each bar represents the mean value of eight fields counted.

To ascertain the effect on invasion of ECE-1 isoform expression in the epithelial compartment, PC-3 cells were transiently transfected with ECE-1a, ECE-1b, ECE-1c or ECE-1d, and invasion was subsequently measured using the matrigel invasion assay. The invasion of PC-3 cells transfected with ECE-1a decreased by approximately 50% (relative to untreated control) in the presence of untreated stromal cells, whereas the expression of ECE-1c significantly increased PC-3 cell invasion by approximately 40% in the presence of untreated stromal cells (Figure 2A). Transient transfection of PC-3 cells with either ECE-1b or ECE-1d did not significantly alter the invasion (Figure 2A).

To determine the effect of ECE-1 isoform expression in the stromal compartment, ECE-1a ECE-1b, ECE-1c or ECE-1d isoforms were expressed in primary prostate stromal cells, and invasion of PC-3 cells was subsequently measured in coculture. Prostate cancer-3 cell invasion in the presence of ECE-1a expressing stroma decreased by approximately 25%, but no significant change was observed for the other isoforms (Figure 2B). As only ECE-1a and ECE-1c significantly altered the invasion, all subsequent experiments were carried out using these isoforms.

### Endothelin-converting enzyme 1a isoform abrogates the influence of ECE-1c isoform in cotransfection

**Figure 3:**
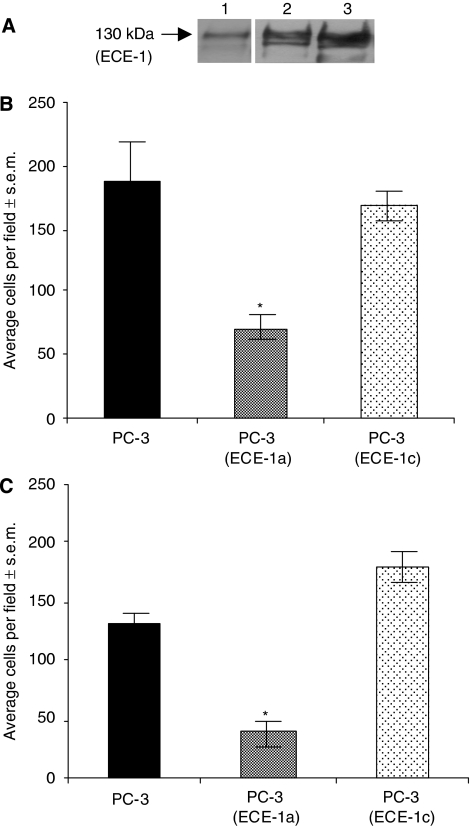
The effect on invasion of ECE-1 isoforms expressed concomitantly in the epithelial and stromal compartment. The PC-3 cells were transfected with ECE-1a and ECE-1c isoforms using FuGENE-6. Exogenous ECE-1 expression levels were determined using (**A**) western blot analysis (monoclonal ECE-1 antibody); lane 1, PC-3 cells; lane 2, PC-3 cells expressing ECE-1a; lane 3, PC-3 cells expressing ECE-1c. These cells were then used in a matrigel invasion assay in the presence of stroma transfected with (**B**) ECE-1a or (**C**) ECE-1c. Each bar represents the mean value of eight fields counted; ^*^P<0.05.

To determine the effect of parallel ECE-1 isoform expression in the epithelia and stroma, PC-3 cells were transfected with either ECE-1a or ECE-1c, and their invasion was subsequently measured in the presence of ECE-1a expressing stroma (Figure 3A). Endothelin-converting enzyme 1a stroma in coculture with ECE-1a epithelia significantly reduced cell invasion by approximately 65% (more than in the presence of untreated stroma (Figure 2A)). Endothelin-converting enzyme 1a stroma in coculture with ECE-1c epithelia counter suppresses the cell invasion resulting in overall invasion levels similar to control. The expression of ECE-1c in the stroma had no effect on the invasion of ECE-1a or ECE-1c-transfected PC-3 cells (Figure 3B).

### Endothelin-converting enzyme 1c isoform can transform low-invasive cells to cells with a highly invasive phenotype

**Figure 4:**
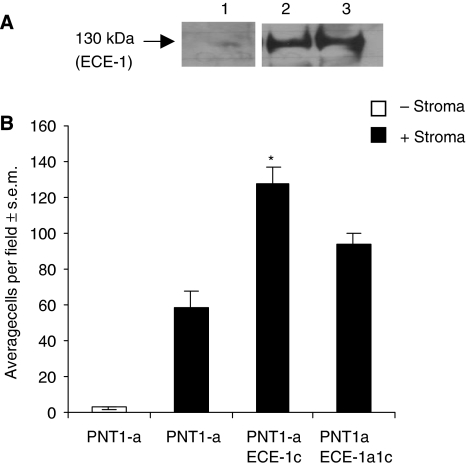
The suppressive effect of ECE-1a isoform on invasion-promoting properties of ECE-1c in the presence of stroma. PNT1-a cells were transfected with the ECE-1c isoform using FuGENE-6. Exogenous ECE-1 expression levels were determined using (**A**) western blot analysis (monoclonal ECE-1 antibody); lane 1, PNT1-a cells; lane 2, PNT1-a cells expressing ECE-1a; lane 3, PNT1-a cells expressing ECE-1c. These cells were then used in a matrigel invasion assay in the presence of stroma. Each bar represents the mean value of eight fields counted; ^*^P<0.001.

PNT1-a, a low-invasive cell line (Lang *et al*, 2000; Dawson *et al*, 2004), which expresses negligible ECE-1 (Dawson *et al*, 2004) was transiently transfected with the ECE-1 isoforms, and invasion through matrigel was measured. Transient expression of ECE-1c in PNT1-a cells increased invasion by 100% in the presence of untreated stromal cells, compared with untransfected PNT1-a (Figure 4). Cotransfection of ECE-1a and ECE-1c into PNT1-a cells reduced this invasive margin to approximately 30 % (Figure 4).

### Conditioned media alone from ECE-1a expressing stromal cells can decrease cell invasion

**Figure 5:**
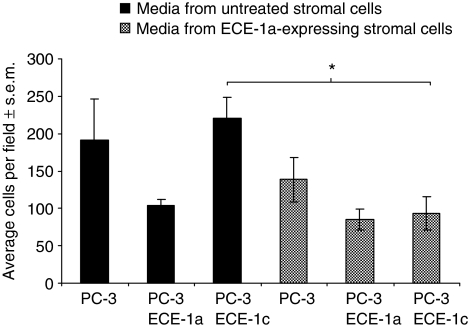
The effect of conditioned media from ECE-1a expressing stromal cells on PC-3 invasion. Stromal cells were transfected with ECE-1a using FuGENE-6 and incubated for 48 h. The media from untreated stroma and ECE-1a expressing stroma was collected and added to the lower well of the invasion chamber. Prostate cancer-3 cells were transfected with ECE-1a and ECE-1c, incubated for 48 h and added to the upper well of the invasion assay. Each bar represents the mean value of eight fields counted.

To investigate whether the effects on cell invasion were mediated by a soluble agent, the invasion of ECE-1a- and ECE-1c expressing PC-3 cells was assessed using either media from normal stromal cells or conditioned media from stromal cells transfected with ECE-1a for 48 h. In the presence of normal stromal media, the invasion of ECE-1a expressing PC-3 cells decreased by approximately 40% (relative to control), whereas the invasion of ECE-1c expressing PC-3 cells increased by approximately 20% (Figure 5). In the presence of conditioned (ECE-1a) stromal media, the invasion of ECE-1a expressing PC-3 cells further decreased by approximately 50% (relative to control). Interestingly, the invasion of ECE-1c expressing PC-3 cells also decreased by approximately 40%, indicating the likely presence of a soluble invasion-suppressing factor in the conditioned media.

## Discussion

Elevated ECE-1 levels are increasingly being correlated with tumour progression in PC and other types of malignancies (Egidy *et al*, 2000b; Arun *et al*, 2001; Dawson *et al*, 2004, 2006; Awano *et al*, 2005; Smollich *et al*, 2007). We have previously recognised the ability of ECE-1 to promote PC cell invasion *in vitro* (Dawson *et al*, 2004). A specific chemical inhibitor of endogenous ECE-1 activity in stromal cells significantly reduced the ability of PC-3 cells to invade through matrigel as a consequence of reduced levels of ET-1 peptide (Dawson *et al*, 2004). With respect to the four recognised isoforms of ECE-1 (ECE-1a-d), ECE-1c is the only isoform expressed in PC cells, with increased expression in metastatic, androgen-independent cells (Dawson *et al*, 2006). This study identified novel roles for two of the ECE-1 isoforms (ECE-1a and ECE-1c) in the invasion process.

Initially, siRNA duplexes (non-isoform specific) targeted to stromal or epithelial ECE-1 were successfully used to reduce invasion with a dramatically greater reduction, if both compartments were targeted simultaneously. Addition of exogenous ET-1 only partially recovered this effect on invasion implicating an alternative role for ECE-1 independent of ET-1 generation. A unique role for ECE-1 has also been proposed by Berger *et al* (2005) who demonstrated that ECE-1 inhibitors could inhibit proliferation of human glioblastoma cells without reducing ET-1 levels (Berger *et al*, 2005). Endothelin-converting enzyme 1a and ECE-1c isoforms were next transiently expressed in epithelial (PC-3) and stromal (STO) cells to assess their influence on invasion. Endothelin-converting enzyme 1c was the sole invasion-promoting isoform, with overexpression resulting in a further increase in the invasive capacity of PC-3 cells. More importantly, this effect was reproducible with ECE-1c expressed either in the epithelia or the stroma, indicating both an autocrine and paracrine influence. Transient expression of ECE-1a, which is normally absent from PC-3 and stromal cells, suppressed the invasion of PC-3 cells. As with ECE-1c, this effect was also reproducible in both epithelial and stromal compartments. More importantly, the expression of ECE-1a in the stromal compartment counteracted the invasion-promoting properties of ECE-1c expression in PC-3 cells, reflecting a property normally associated with suppressor proteins, such as PTEN, p53 and NEP (Li *et al*, 1997; Sumitomo *et al*, 2004).

Although ECE-1 has been reported in a number of cancers (Ahmed *et al*, 2000; Egidy *et al*, 2000a, 2000b; Arun *et al*, 2001; Usmani *et al*, 2002; Dawson *et al*, 2004, 2006; Awano *et al*, 2005; Smollich *et al*, 2007), specific isoforms present have yet to be identified. To our knowledge, ECE-1a expression has not been reported in any cancer cells either *in vitro* or *in vivo*, and only ECE-1c is abundant in prostate and lung cancer (Ahmed *et al*, 2000; Dawson *et al*, 2006). Given that the expression of each isoform is regulated by distinct promoters, it is likely that different stimuli may regulate each isoform differently. This hypothesis is supported by the work of Orzechowski *et al* (2001) who demonstrated that PKC activation by treatment with phorbol ester resulted in the upregulation of ECE-1a expression, but had no effect on the other ECE-1 isoforms (Orzechowski *et al*, 2001). This regulation was at the level of transcription involving the transcription factor *Ets*-1, suggesting that the binding site for this factor may not be present in the regulatory region of the other isoform promoters.

Interestingly, in the presence of conditioned media from ECE-1a-transfected stromal cells, the invasion of PC-3 cells was compromised. Furthermore, ECE-1a-conditioned media also counteracted the invasive ability of ECE-1c-transfected PC-3 cells, suggesting the possibility of a soluble factor in the conditioned media. One possibility is that it might be a soluble form of ECE-1a in the media. A soluble secreted form of ECE-1 protein has recently been reported (Kuruppu *et al*, 2007). Endothelin-converting enzyme 1, expressed endogenously in human umbilical vein endothelial cells, is subject to constitutive ectodomain shedding, although the secretase responsible is not identified (Kuruppu *et al*, 2007).

Alternatively, it is possible that the secreted ECE-1a can form a heterodimer with ECE-1c at the cell surface, initiating the internalisation of the ECE-1a/ECE-1c complex. Endothelin-converting enzyme 1b has already been shown to form heterodimers (Muller *et al*, 2003) with other ECE-1 isoforms, and acts as a vehicle for regulating their distribution (Muller *et al*, 2003). Given the known signalling capability of NEP, a close homologue of ECE-1, it can be speculated that heterodimerisation of ECE-1 isoforms may trigger intracellular signalling. A similar mechanism exists for other cell surface proteins, such as members of the ErbB family (Zhan *et al*, 2006).

In conclusion, the exploitation of the ECE-1 isoforms may lead to a new generation of molecular-targeted therapies for prostate and other cancers.
